# The Use of Qualitative Behaviour Assessment for the On-Farm Welfare Assessment of Dairy Goats

**DOI:** 10.3390/ani8070123

**Published:** 2018-07-19

**Authors:** Monica Battini, Sara Barbieri, Ana Vieira, Edna Can, George Stilwell, Silvana Mattiello

**Affiliations:** 1 Laboratorio di Benessere animale, Etologia Applicata e Produzioni Sostenibili, Dipartimento di Medicina Veterinaria, Università degli Studi di Milano, Via G. Celoria 10, 20133 Milan, Italy; sara.barbieri@unimi.it (S.B.); silvana.mattiello@unimi.it (S.M.); 2 Instituto Superior Técnico, Centre for Management Studies, Universidade de Lisboa, Avenida Rovisco Pais, 1049-001 Lisboa, Portugal; ana.lopesvieira@gmail.com; 3 Division of Agricultural and Environmental Sciences, Sutton Bonington Campus, University of Nottingham, College Road, Sutton Bonington, Loughborough LE12 5RD, UK; ednacan@gmail.com; 4 Faculdade de Medicina Veterinária, Universidade de Lisboa, Polo Alto da Ajuda, 1300-477 Lisbon, Portugal; stilwell@fmv.ulisboa.pt

**Keywords:** dairy goats, Qualitative Behaviour Assessment, emotion, welfare assessment protocol, welfare indicators

## Abstract

**Simple Summary:**

The use of feasible indicators to evaluate animals’ emotional states in farm animals is strongly encouraged for welfare assessment. The inclusion of qualitative behaviour assessment (QBA) in on-farm protocols has been constantly increasing during the last few years; but its association with other welfare measures has been scarcely investigated so far. In the present study; we investigated whether QBA shows a meaningful coherence with other measures included in the AWIN (Animal Welfare Indicators) welfare assessment protocol for dairy goats. We confirmed that QBA can clearly discern mood (from Agitated/Alert to Content/Relaxed) and the level of activity (from Bored to Lively) in goats. Furthermore; goats with a shiny hair coat seem more relaxed and sociable than goats with a poor hair coat condition. In contrast; farms where the workload for the stockperson is high have goats that were observed as more bored and suffering; probably because farmers do not invest enough time in taking care of their animals. Even though we found only few relations between QBA and the other measures of the AWIN welfare assessment protocol; the holistic approach of QBA can be useful to integrate the assessment and give a different perspective on the complexity of animals’ emotions and overall welfare state.

**Abstract:**

This research investigated whether using qualitative behaviour assessment (QBA) with a fixed list of descriptors may be related to quantitative animal- (ABM) and resource-based (RBM) measures included in the AWIN (Animal Welfare Indicators) welfare assessment prototype protocol for goats, tested in 60 farms. A principal component analysis (PCA) was conducted on QBA descriptors; then PCs were correlated to some ABMs and RBMs. Subsequently, a combined PCA merged QBA scores, ABMs and RBMs. The study confirms that QBA can identify the differences in goats’ emotions, but only few significant correlations were found with ABMs and RBMs. In addition, the combined PCA revealed that goats with a normal hair coat were scored as more relaxed and sociable. A high farm workload was related to bored and suffering goats, probably because farmers that can devote less time to animals may fail to recognise important signals from them. Goats were scored as sociable, but also alert, in response to the presence of an outdoor run, probably because when outdoors they received more stimuli than indoors and were more attentive to the surroundings. Notwithstanding these results, the holistic approach of QBA may allow to register animals’ welfare from a different perspective and be complementary to other measures.

## 1. Introduction

Qualitative behaviour assessment (QBA) is a scientific method that relies on the ability of human observers to integrate perceived details of behaviour, posture, and context into the summarization of animals’ style of behaving, using descriptors such as “relaxed”, “tense”, “frustrated”, or “content” [1]. The innovation in this approach stems from translating the emotion of animals judged by the observers into figures that a formal statistical methodology can analyse [2]. After observing the animals, observers give a score on a visual analogue scale (VAS) for each QBA descriptor [3]. Terms used to describe animals’ demeanour can either be generated by each observer (free choice profiling method [1]) or compiled beforehand for all observers (fixed list method [4]). The latter is a preferable method to be used on farm for feasibility reasons (e.g., no complex “term-finding-phase” required).

Thus far, QBA has been used for a wide range of species in different contexts, but only a limited number of these studies applied QBA in on-farm conditions using a fixed list of terms (e.g., layers and broilers [5]; cattle [4,6]; pigs [7]; buffaloes [8]; veal calves [9]; horses [10]; dairy goats [11]; donkeys [12]; and sheep [13]).

The inclusion of QBA in welfare assessment protocols has been constantly increasing, first of all because QBA may capture also positive aspects of animal welfare, rather than focus only on negative aspects. Furthermore, the holistic approach goes beyond the application of single traditional quantitative indicators (e.g., body condition score, lameness), and allows to draw attention to the complexity of the animals’ demeanour and to identify differences in animals’ emotions expressed as valence and arousal [2]. Finally, it is feasible on a farm, since no specific equipment is required and is less time-consuming when compared to the recording of other positive indicators (e.g., play and affiliative behaviour [14]).

To our knowledge, few attempts have been made to associate QBA results using a fixed list of terms with other animal-based or resource-based measures. This seems particularly important for on-farm use, where there is a risk of observers being biased by the farm environment. Phythian et al. [13] found that the presence of lame and dull sheep was associated with negative mood scores, while QBA scores on responsiveness showed a weak correlation with breech soiling. Positive QBA descriptors were found to be associated with positive human-donkey interaction indicators (absence of tail tuck, no avoidance, and positive reaction to an assessor walking down the side of the donkey [12]). In horses, “explorative/sociable” attributes were found to be associated with animals engaging in close contact with humans, while “suspicious/nervous” attributes were recorded in horses showing immobility behaviour [10]. The dark period length in broiler farms was associated with different levels of arousal in interaction with the environment [15]. Andreasen et al. [6] only found weak correlations with QBA and welfare Criteria, Principles, and the overall Welfare Quality^®^ assessment for dairy cattle.

After validation of a fixed list of terms specifically developed for dairy goats [11], QBA was included in the Animal Welfare Indicators (AWIN) welfare assessment protocol for goats [16].

The present research aimed at investigating whether a fixed QBA rating scale developed for adult dairy goats may be related to other quantitative animal-based and resource-based measures collected at the same time during the application of the AWIN welfare assessment prototype protocol for goats.

## 2. Material and Methods

Thirty commercial dairy goat farms in Northern Italy and 30 in Portugal (mean ± SEM, Standard Error of the Mean) farm size: 266.00 ± 44.43 adult goats/farm) were visited between February and July 2014. All farms kept goats permanently indoors on straw litter and 70% of farms also had an outdoor run for daily exercise (mean ± SEM of free access days to outdoor run: 299.22 ± 14.05; mean ± SEM of accessible hours to outdoor run: 18.29 ± 1.26). Breeds were mainly Saanen and Alpine, but 38% of farms exclusively bred Murciana or Anglo-Nubian goats. Goats were milked twice a day and fed with total mixed ration (forages and concentrate). During the day, feed was distributed 2.29 ± 0.12 times (mean ± SEM); roughage was always available in 85% of farms, whereas only 8.3% of farms left concentrate always available. The individual feeding space was 0.45 ± 0.06 m (mean ± SEM). Water was permanently available. Farmers were mainly males (81.7%; mean ± SEM age: 41.85 ± 1.63 years) and the workload was 121.75 ± 15.98 goats/worker (mean ± SEM).

The AWIN prototype protocol was applied on all the 60 farms by the same single observer per country [17]. Observers received a common training on the whole prototype protocol, including the description and application of each indicator and the order of data collection (Table 1) [17].

Preliminary studies revealed a good inter-observer reliability (on PC1, r = 0.910 and PC2, r = 0.906; *p* = 0.001) [18] of adequately trained observers.

The collection of all the welfare indicators was performed on a single pen (mean ± SEM pen size: 72.28 ± 7.40 lactating goats; mean ± SEM space availability: 2.41 ± 0.27 m^2^/goat), based on the criteria setup in the prototype protocol. Criteria and sampling strategy are described in Battini et al. [17]. QBA observations were always performed on the whole group in the pen at least 30 min after feed distribution. It is recommended that QBA is performed in periods when animals can express different behaviours and be engaged in various activities. As confirmed in previous research, 30 min after feed distribution, only a few goats are still at the feeding rack. QBA observation sessions lasted from 10 to 20 min, from one to three observation points, depending on the pen size and structure (for details see [11,16]). If a pen had an outdoor run, both indoor and outdoor areas were observed. It is mandatory that during QBA observations no other indicator is collected. At the end of the QBA observation session, the assessor found a quiet spot far from the surveyed pen (out of reach and sight of the animals) to score the goats using the list of QBA descriptors and respective definitions deriving from the refinement of the list used by Grosso [11] and reported in Table 2.

A principal component analysis (PCA, correlation matrix, no rotation) was conducted on QBA descriptors. As the distribution of both quantitative animal-based (ABMs) and resource-based welfare measures (RBMs) was not normal (according to Kolmogorov-Smirnov test), the relationship of QBA scores with ABMs and RBMs collected in all the 60 farms was investigated using appropriate non-parametric tests: Spearman’s rank test (ρ) for continuous variables or non-parametric ANOVA (Kruskal-Wallis test) for binary variables (e.g., presence of outdoor run). The selected ABMs presented sufficient variability in our sample and, in previous studies on other species, they appeared to be related to the emotional state (severe lameness and overgrown claw [13]; Acceptance, as an indicator of the quality of human-animal relationship during avoidance distance test [10,12]), or were specifically related to the general health status of goats (udder asymmetry, hair coat condition, abscesses, body condition score (BCS) [19,20,21]. Quantitative ABMs were expressed as the proportion of animals with no welfare problems, calculated at pen level, as indicated in the AWIN protocol for dairy goats [16]. Space availability, individual feeding space, presence of outdoor run and workload were included as RBMs.

A second PCA analysis (combined) was conducted merging QBA scores with the selected ABMs and RBMs.

## 3. Results

The proportion of animals with no welfare problems for relevant quantitative indicators calculated at pen level is reported in Table 3.

The PCA conducted on QBA descriptors identified four main components (eigenvalues: 3.53, 1.92, 1.37 and 1.29 for PC1, PC2, PC3, and PC4, respectively), explaining 62.43% of the variation between farms (27.16, 14.75, 10.55, 9.96 for PC1, PC2, PC3, and PC4, respectively).

On PC1, QBA descriptors ranged from content/relaxed to agitated/alert, describing how the animals are at ease with the environment. On PC2, descriptors range from curious/lively to bored and better describe emotions connected to the quality of the environment and the possibility to interact with it. The first two PCs seem more interesting in drawing a picture of goats’ emotions and level of activities than the third and fourth PCs. Descriptors on PC3 are not clearly distributed: they range from sociable/suffering to content/relaxed (with very low loadings). On PC4, descriptors range from fearful to frustrated.

Correlations among PCs and ABMs and RBMs are presented in Table 4.

Significant correlations were only found for normal hair coat (*p* = 0.030) with PC1 and Workload and individual feeding space (both *p* = 0.001) with PC3.

The combined PCA conducted merging QBA scores, ABMs and RBMs identified four main components (eigenvalues: 3.80, 2.45, 2.31, and 1.96 for PC1, PC2, PC3, and PC4, respectively), explaining 43.83% of the variation between farms (15.82, 10.22, 9.63, 8.16 for PC1, PC2, PC3, and PC4, respectively).

No differences in PC loadings were recorded depending on the presence/absence of an outdoor run, although the difference on PC4 approached statistical significance (*p* = 0.090).

**Figure 1:**
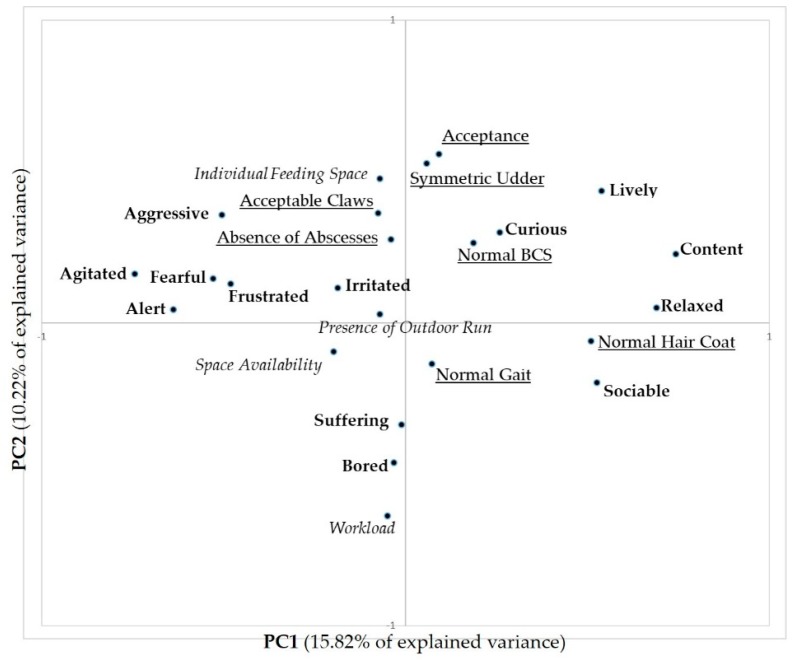
Loadings of Qualitative Behaviour Assessment descriptors (in bold), other quantitative animal-based measures (underlined), and resource-based measures (in italics) along the first two PCs for the 60 farms.

Figure 1 shows the distribution of QBA descriptors and all the variables included along the first two PCs (total explained variance 26.04%).

On PC1, QBA descriptors explain the emotional valence of goats, ranging from agitated and alert (negative mood, on the left) to content and relaxed (positive mood, on the right). PC2 explains the level of activity of goats, ranging from bored (low level on the bottom) to lively (high level on the top).

As to the relationship of QBA descriptors with other indicators, a positive and relevant loading on PC1 (positive mood) was observed for the ABM normal hair coat (0.507). No RBM showed a high weight on PC1. Among ABMs, the highest positive loadings on PC2 were obtained by symmetric udder (0.530) and acceptance (0.560), whereas among RBMs workload (−0.633) had the highest negative weight, and individual feeding space (0.480) had the highest positive weight (Figure 1).

**Figure 2:**
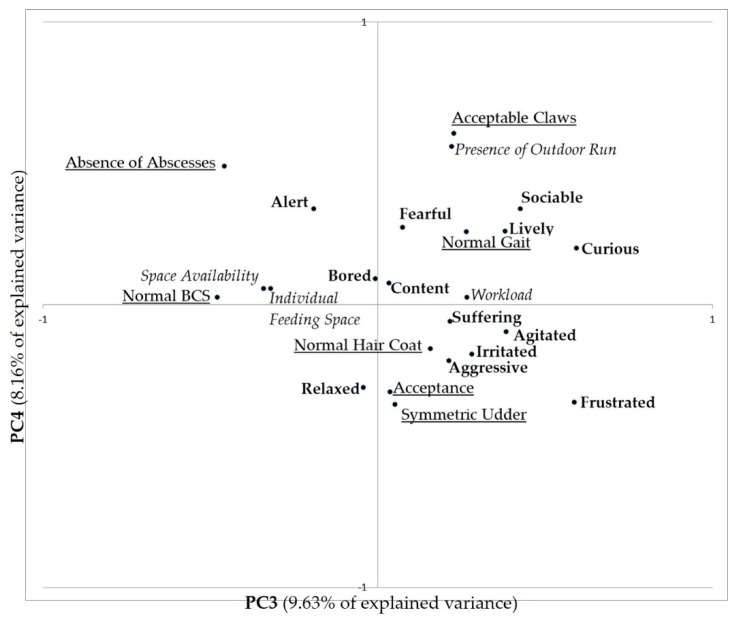
Loadings of QBA descriptors (in bold), other quantitative animal-based measures (underlined), and resource-based measures (in italics) along the third and fourth PCs for the 60 farms.

Figure 2 shows the distribution of QBA descriptors and all the variables included along the third and fourth PCs (total explained variance 17.79%).

On PC3, high loadings were observed for Normal BCS (−0.480) and absence of abscesses (0.459), with no clear relationship with the emotional state that ranges from curious to alert. On PC4 we can observe a weak tendency towards a more positive mood on the top, associated to high levels of acceptable claws (0.608), absence of abscesses (0.491), and to the presence of an outdoor run (0.562).

## 4. Discussion

The present study confirms that QBA can provide interesting details on the way that goats interact with their environment and the related emotions (Table 5). This is particularly evident along the first two PCs that describe how much animals feel at ease with the environment and how much the environment can be stimulating. However, only few significant correlations were found with other quantitative measures. Normal hair coat showed a significant, although weak, correlation with PC1: animals that have higher scores of content and relaxed also have the hair coat in good condition. This correlation is confirmed by the results of the combined PCA conducted merging QBA descriptors and other quantitative measures (Figure 1), where normal hair coat reveals an interesting relation with positive moods. Goats with normal hair coats (defined as shiny, homogeneous, and adherent to the body [16]) had a higher score of relaxed and sociable descriptors. Hair coat condition encompasses different aspects of welfare. Battini et al. [19] found that a poor hair coat condition (defined as uneven, shaggy, matted, rough, or scurfy hair coat, frequently longer than normal) in goats is significantly associated with mineral deficiencies or surplus, poor body condition, and abnormal lung sounds, and is possibly related to chronic diseases. Therefore, we can expect that goats with poor hair coats show a negative emotional state, whereas goats with normal hair coats are more likely to have a positive mood that in our study is shown by positive interactions with other goats (high values of Sociable) and feeling at ease with the environment (high values of relaxed and content).

The other significant correlations found with PC3 and workload and individual feeding space are more difficult to explain. However, the results of the combined PCA can be useful to interpret the results obtained with correlations. An interesting negative weight of workload can be observed on PC2, corresponding to high loadings of bored and suffering (Figure 1), and workload is also positively correlated to suffering on PC3 (Figure 2). We can suppose that when workload is high, farmers devote less time to animals and miss the possibility to identify important signals from them. Furthermore, a high workload resulted in a significant risk factor for a negative human-animal relationship, as indicated by a reduced acceptance and contact of goats with humans [22].

The fact that the presence of outdoor run has such a low weight on both PC1 and PC2 seems in contrast with findings by Grosso et al. [11], showing that goats kept indoor have more negative moods (e.g., “aggressive”, “irritated”, and “suffering”) than goats kept outdoor. However, we have to take into account that the outdoor area in Grosso’s study was represented by pasture, whereas in the present study the outdoor area, when present, was often represented only by an outdoor run, often without grass, with variable access characteristics in terms of size (m^2^/goat), daily availability (days of use and hours per day of use) and other characteristics. This suggests that the provision of outdoor runs is not sufficient to positively influence goats’ emotions, but its characteristics, design, and use should probably also be taken into account. The presence of outdoor run seems to be important on PC4, where it is positively related to acceptable claws, but also to goats scored as sociable and alert (with an almost significant effect highlighted by Kruskal-Wallis test). The first relation can confirm that the use of outdoor runs can facilitate the proper wear of claws. As to the emotions, the presence of outdoor run can contribute to increase the social relationships of goats, maybe reducing the aggressive interactions and the frustration in a confined environment (presence of outdoor run is also negatively related to aggressive and frustrated). However, the novel stimuli that can be present outdoors (e.g., sounds, other people, other animals) seem to make goats more alert towards potential dangers and source of perils.

The lack of relation with QBA descriptors and Space availability can be explained by the fact that, on average, farms in the present study had a space availability higher than the suggested values (2.41 m^2^/goat vs. 1.5 m^2^/goat; [23]).

The lack of a relation of acceptance with a positive mood is in contrast with findings from other authors in horses [10] and donkeys [12]. This may be partly explained by the fact that, in horses, QBA observations were performed at the same time of the evaluation of human-animal relationship, whereas in the AWIN protocol the order of data collection required the application of QBA before the evaluation of this relationship. However, this explanation is not completely satisfactory, as in donkeys [12] QBA observations were performed before the evaluation of human-animal relationship, as in our study. On the other hand, in our experiment, Acceptance was positively related to a high level of arousal on PC2, probably due to the fact that curious and lively animals are more prone to approach humans and to accept to get in contact with them, and this seems in line with the observation of explorative horses engaging in close contact with humans [10]. An experimental study conducted by Miller et al. [24] confirmed that goats subjected to high degree of positive human interactions were scored by observers as more “calm/content” compared to goats that received a low degree of interactions, scored as more “agitated/scared”. Goats that were more habituated to humans also obtained scores negatively correlated with the number of agonistic contacts and flight speed. The results of this study are interesting, but the trial was conducted in an experimental setting, thus, in a situation that was very different from that of the present research and of the above mentioned studies conducted in horses and donkeys [10,12] that were conducted on farms. First, the study conducted by Miller et al. [24] used a free choice profile method for term generation and not the fixed list, and QBA observations were conducted watching video clips and not by direct observations. Furthermore, studies involving QBA generally ground on contrasting expressive qualities where treatments are previously selected for their divergent characteristics (as in Miller et al. [24]), but this may unlikely happen in on-farm studies [6].

Other ABMs with a positive weight on PC2 in the combined PCA analysis are acceptable claws, absence of abscesses, symmetric udder and normal BCS. Acceptable claws can actually be related to a higher possibility of moving and being active, and this explains the relation of this indicator with a high level of arousal, whereas the other three measures are related to a better health condition, that is also supposed to be related with a high level of vitality and activity. This is also in agreement with the results of previous research showing that too thin goats had fertility problems [25] and higher mortality rates [26], suggesting a lower vitality and, therefore, a low level of arousal. However, none of these measures showed a significant correlation with the PCs generated by PCA analysis of QBA descriptors.

On PC4, normal gait seems positively related to goats scored as lively. This is expected as animals that are free to move in the pen can also be seen engaged in different activities. Lameness being one of the most prevalent welfare issues in goat farms [27], this finding is of particular interest as it confirms that the absence of pain due to lameness or of difficulties in moving around the environment can positively influence the emotions and the level of activities of goats during the day.

The present research only investigated the relation with some ABMs and RBMs included in the AWIN welfare assessment prototype protocol. Therefore, these results are influenced by the selection of the indicators included in the protocol and further meaningful relations might be found with other quantitative measures. For example, in other studies correlations were found with many physiological measures [28,29,30,31], but our protocol did not include any physiological variable due to feasibility reasons. Some correlations were also observed in other species with behavioural measures, such as social, vigilance, and vocal behaviour [10,31], but in the AWIN protocol only one behavioural indicator (Acceptance) was included, for the above mentioned feasibility reasons. Our protocol included mainly clinical indicators, but, in line with the findings of other authors [13,28], who found only few, and weak, correlations between clinical variables and QBA, only hair coat condition was clearly associated with QBA outcomes. So far, the influence that RBMs can have on goats’ emotions is commonly neglected. Our study revealed the interesting effect that workload can have on goats’ emotions, confirming the importance of spending time with animals and at the same time highlights some of the biggest problems reported by farmers in commercial farms: the lack of time and the excessive workload to have a good quality of work.

## 5. Conclusions

QBA can show clear differences in valence and activity level of dairy goats. However, in spite of some interesting relations with hair coat condition and workload, our results show that there are few relations between QBA and the quantitative animal-based and resource-based measures included in the AWIN protocol for dairy goats.

Notwithstanding these few relations, QBA, being a whole-animal indicator, may register animals’ welfare from a different perspective and not be necessarily directly linked to other indicators included in a welfare assessment protocol; QBA can be considered as a complement to other measures for assessing the animals’ overall welfare state.

Although the emotional state cannot always be directly linked to specific welfare issues, assessors can use the results of QBA to promote a discussion with farmers about the mood and level of activity of their animals, thus fostering farmers’ awareness of the complexity of animal welfare.

## Figures and Tables

**Table 1:** Order of collection and duration of the time of data collection of the indicators included in the prototype of the AWIN (Animal Welfare Indicators) protocol.

Order of Collection	Indicator	Duration
1	Queuing at feeding	15 min
2	Queuing at drinking	15 min
3	Hair coat condition	10 min
4	Improper disbudding	
5	Kneeling at the feeding rack	
6	Kneeling in the pen	
7	Oblivion	
8	Abnormal lying	
9	Panting Score	
10	Shivering Score	
11	Qualitative Behaviour Assessment (QBA)	10–20 min
12	Latency to first contact test	max 5 min
13	Avoidance distance test	max 10 min
14	Severe lameness	max 5 min
15	Body Condition Score (BCS)	30–45 s/goat (total duration depends on sample size)
16	Faecal soiling	
17	Vulvar discharge	
18	Udder asymmetry	
19	Cleanliness	
20	Abscesses	
21	Lesions	
22	Overgrown claws	
23	Knee calluses	
24	Ocular discharge	
25	Nasal discharge	

**Table 2:** List of Qualitative Behaviour Assessment descriptors and definitions.

Descriptors	Definitions
Aggressive	An aggressive goat bites other goats (especially the ears), voluntarily attacks or threatens other goats with the intention of hurting or disturbing them, butts the belly or the head of other goats. It is intentionally harmful to other goats. The aggressive behaviour can be related to dominance, fear, or resource protection.
Agitated	An agitated goat is restless, not at ease, highly susceptible to stimuli, it can move her ears, vocalize, or nervously move around.
Alert	An alert goat is on guard against danger, watchful and ready to react to a potential source of peril (e.g., sounds, person, object, animal). It can emit acoustic or visual alarm signals (e.g., vocalizations, snorts, stamping, ears in upright position, stiff body). It often stands motionless, directing its attention towards the potentially negative stimulus.
Bored	A bored goat is wearied, dull, or is uninterested in the surrounding environment (low reactivity); lack of stimulation; it may be looking for something to do.
Content	A content goat is appeased, gratified, happy, comfortable, at ease, satisfied about its environment, playful. It may jump, play and make noise with objects, climb, or try to climb.
Curious	A curious goat is reactive, engaged in exploratory behaviour, positively intrigued by something, attracted by the surrounding environment and by novelties (e.g., people, goats in oestrus, objects). It looks around, but often concentrates its gaze in a specific direction or towards a signal, which attracts its interest.
Fearful	A fearful goat is a scared and shy animal. It may look for shelter or for a way out and crouches down or may tend to hide in the middle of the group. There may be a whole group running around.
Frustrated	A frustrated goat is annoyed and impatient because it is prevented from achieving something (e.g., queuing at the feeding rack or at the water places, passive behaviour).
Irritated	An irritated goat is bothered or annoyed by something (e.g., flies, pruritus, noise, another goat) that can disturb, upset, trouble, or exasperate it.
Lively	A lively goat is active, busy and positively engaged in different activities, full of life and expressing energy.
Relaxed	A relaxed goat is at ease in the surrounding environment.
Sociable	A sociable goat is friendly to other goats. It has affiliative (e.g., grooming, sniffing, resting in pairs) and playful contacts with other goats.
Suffering	A suffering goat is enduring pain, often with contracted muscles, possibly in antalgic postures. It frequently shows little or no movement or reaction to stimuli and often remains isolated from the group.

**Table 3:** The proportion of animals with no welfare problems for the selected animal-based quantitative measures calculated at the pen level.

Welfare Measure	Min–Max	Mean	Standard Error of the Mean	Standard Deviation
Normal Hair Coat (%)	30–100	75.95	2.15	16.63
Acceptance (%)	0–37.50	4.14	0.91	7.06
Normal gait (%)	84–100	97.43	0.46	3.58
Normal BCS (%)	33.33–100	79.34	1.83	14.15
Symmetric udder (%)	80–100	95.49	0.64	4.94
Absence of abscesses (%)	6.67–100	78.45	2.61	20.24
Acceptable claws (%)	4.17–100	59.82	4.00	30.96

**Table 4:** Correlations between the first four Principal Components (PCs) and the selected ABMs and RBMs. Statistical differences are bold typed.

Principal Component	Normal BCS	Symmetric Udder	Absence of Abscesses	Acceptable Claws	Normal Hair Coat	Acceptance	Normal Gait	Workload	Space Availability	Individual Feeding Space
PC1	ρ = 0.08 *p* = 0.55	ρ = 0.00 *p* = 0.98	ρ = 0.01 *p* = 0.93	ρ = −0.02 *p* = 0.90	ρ = 0.288 ***p* = 0.03**	ρ = −0.02 *p* = 0.87	ρ = 0.00 *p* = 0.97	ρ = −0.01 *p* = 0.91	ρ = 0.06 *p* = 0.63	ρ = 0.06 *p* = 0.67
PC2	ρ = 0.04 *p* = 0.78	ρ = 0.23 *p* = 0.07	ρ = −0.09 *p* = 0.51	ρ = 0.21 *p* = 0.11	ρ = 0.23 *p* = 0.08	ρ = 0.14 *p* = 0.27	ρ = 0.22 *p* = 0.10	ρ = −0.07 *p* = 0.57	ρ = −0.05 *p* = 0.68	ρ = 0.14 *p* = 0.28
PC3	ρ = −0.22 *p* = 0.09	ρ = −0.06 *p* = 0.64	ρ = −0.07 *p* = 0.61	ρ = 0.05 *p* = 0.71	ρ = 0.05 *p* = 0.68	ρ = −0.08 *p* = 0.56	ρ = 0.07 *p* = 0.59	ρ = 0.368 ***p* = 0.00**	ρ = −0.07 *p* = 0.59	ρ = −0.367 ***p* = 0.00**
PC4	ρ = 0.14 *p* = 0.30	ρ = −0.01 *p* = 0.97	ρ = 0.19 *p* = 0.14	ρ = 0.15 *p* = 0.25	ρ = 0.17 *p* = 0.20	ρ = −0.14 *p* = 0.29	sρ = 0.13 *p* = 0.33	ρ = 0.01 *p* = 0.92	ρ = −0.07 *p* = 0.61	ρ = 0.03 *p* = 0.81

**Table 5:** Loadings for Qualitative Behaviour Assessment descriptors on the first four PCs. The highest loadings for each factor are inbold.

Descriptor	PC1	PC2	PC3	PC4
Aggressive	**−0.578**	**0.424**	−0.138	−0.028
Agitated	**−0.770**	**0.405**	0.001	−0.164
Alert	**−0.625**	−0.066	−0.144	**0.487**
Bored	0.004	**−0.418**	**0.525**	−0.248
Content	**0.717**	0.314	−0.347	0.170
Curious	0.222	**0.700**	0.321	0.282
Fearful	**−0.530**	0.163	−0.026	**0.631**
Frustrated	**−0.517**	**0.507**	0.256	**−0.482**
Irritated	−0.222	0.261	−0.119	−0.356
Lively	**0.537**	**0.605**	0.033	0.011
Relaxed	**0.683**	0.112	−0.304	−0.110
Sociable	**0.544**	0.245	**0.588**	0.128
Suffering	−0.056	−0.129	**0.560**	0.275
